# Supplementary material for: Structural insights into the assembly and polyA signal recognition mechanism of the human CPSF complex
Source: eLife. 2017 Dec 23;6:e33111. doi: 10.7554/eLife.33111 (PMC5760199; doi:10.7554/eLife.33111)
Supplement: Transparent reporting form [file elife-33111-transrepform.docx]

***eLife’s* transparent reporting form**

We encourage authors to provide detailed information *within their submission* to facilitate the interpretation and replication of experiments. Authors can upload supporting documentation to indicate the use of appropriate reporting guidelines for health-related research (see [EQUATOR Network](http://www.equator-network.org/%20)), life science research (see the [BioSharing Information Resource](https://biosharing.org/)), or the [ARRIVE guidelines](http://www.plosbiology.org/article/info:doi/10.1371/journal.pbio.1000412) for reporting work involving animal research. Where applicable, authors should refer to any relevant reporting standards documents in this form.

If you have any questions, please consult our Journal Policies and/or contact us: [editorial@elifesciences.org](mailto:editorial@elifesciences.org).

**Sample-size estimation**

- You should state whether an appropriate sample size was computed when the study was being designed
- You should state the statistical method of sample size computation and any required assumptions
- If no explicit power analysis was used, you should describe how you decided what sample (replicate) size (number) to use

Please outline where this information can be found within the submission (e.g., sections or figure legends), or explain why this information doesn’t apply to your submission:

No explicit power analysis was used.

**Replicates**

- You should report how often each experiment was performed
- You should include a definition of biological versus technical replication
- The data obtained should be provided and sufficient information should be provided to indicate the number of independent biological and/or technical replicates
- If you encountered any outliers, you should describe how these were handled
- Criteria for exclusion/inclusion of data should be clearly stated
- High-throughput sequence data should be uploaded before submission, with a private link for reviewers provided (these are available from both GEO and ArrayExpress)

Please outline where this information can be found within the submission (e.g., sections or figure legends), or explain why this information doesn’t apply to your submission:

The fluorescence polarization assays reported in Figure 1A, Figure 4 and Figure 4-Figure Supplement 1A were performed as follows:

- Each FP measurement at a single concentration (one data point in the graph) was repeated five consecutive times

- Each experiment (measurements of FP at different protein concentrations and fitting of the data) was repeated at least three times (with the exception of CPSF160-WDR33^N^-CPSF30 full-length-Fip1 full-length binding to mutant PAS RNA, which was repeated two times) using the same stock of purified protein (biological sample) that was independently diluted at the appropriate concentrations for each of the three experiments.

For the mass spectrometry experiments (Figure 1B, Figure 1 Figure Supplement 1B and Figure 1 Source Data 1), given that the choice of ion precursor for fragmentation in the mass spectrometer is stochastic, we injected the sample (25% of the reaction volume) two times. We then merged (union) the peptide spectrum matches between the two runs.

**Statistical reporting**

- Statistical analysis methods should be described and justified
- Raw data should be presented in figures whenever informative to do so (typically when N per group is less than 10)
- For each experiment, you should identify the statistical tests used, exact values of N, definitions of center, methods of multiple test correction, and dispersion and precision measures (e.g., mean, median, SD, SEM, confidence intervals; and, for the major substantive results, a measure of effect size (e.g., Pearson's r, Cohen's d)
- Report exact p-values wherever possible alongside the summary statistics and 95% confidence intervals. These should be reported for all key questions and not only when the p-value is less than 0.05.

Please outline where this information can be found within the submission (e.g., sections or figure legends), or explain why this information doesn’t apply to your submission:

For fluorescence polarization assays:

- Each data point in the graph and relative error bar represents the mean and standard error of mean (SEM), respectively, obtained from five consecutive measurements.

- Each experiment (measurements of FP at different protein concentrations) was fitted to a one-site binding model accounting for ligand depletion and the equilibrium dissociation constants with the relative standard error of mean (SEM) was obtained in GraphPad Prism 6.

- The equilibrium dissociation constants and relative SEM obtained from three experiments were averaged in GraphPad Prism 6. The final averaged value of mean and SEM is reported in Table 2.

For mass-spectrometry:

The cross-linked peptide spectral matches are scored by a linear discriminant score and the threshold of 30 is chosen by inspection of spectral quality. This threshold corresponds to a decoy database-based calculation of 10% false discovery rate at the identification level calculated according to Walzthoeni, 2012 Nat Methods.

(For large datasets, or papers with a very large number of statistical tests, you may upload a single table file with tests, Ns, etc., with reference to sections in the manuscript.)

**Group allocation**

- Indicate how samples were allocated into experimental groups (in the case of clinical studies, please specify allocation to treatment method); if randomization was used, please also state if restricted randomization was applied
- Indicate if masking was used during group allocation, data collection and/or data analysis

Please outline where this information can be found within the submission (e.g., sections or figure legends), or explain why this information doesn’t apply to your submission:

No group allocation was used.

**Additional data files (“source data”)**

- We encourage you to upload relevant additional data files, such as numerical data that are represented as a graph in a figure, or as a summary table
- Where provided, these should be in the most useful format, and they can be uploaded as “Source data” files linked to a main figure or table
- Include model definition files including the full list of parameters used
- Include code used for data analysis (e.g., R, MatLab)
- Avoid stating that data files are “available upon request”

Please indicate the figures or tables for which source data files have been provided:

Figure 1-Source Data 1 provides the full list of crosslinked peptides identified by mass-spectrometry on which Figure 1B and Figure 1-Figure Supplement 1B are based. The MS raw data and the cross-linking results are available via ProteomeXchange with identifier PXD008122.
